# The Adoption of Medicaid Expansion and Its Association with Stage at Presentation and Overall Survival for Sarcomas

**DOI:** 10.5435/JAAOSGlobal-D-26-00021

**Published:** 2026-10-16

**Authors:** Sarah E. Lindsay, Yee-Cheen Doung, Kenneth R. Gundle, James Hayden, Duncan C. Ramsey

**Affiliations:** From the Oregon Health & Science University, Portland, OR.

## Abstract

**Background:**

Medicaid expansion under the Affordable Care Act is linked to higher rates of early cancer diagnosis and improved survival. This study examines the effect of Medicaid expansion on stage at presentation and 5-year overall survival (OS) for bone and soft tissue sarcomas.

**Patients and methods:**

We analyzed data from the National Cancer Database for patients diagnosed with bone and soft tissue sarcomas in states with and without Medicaid expansion. Patients were stratified into pre-expansion (2010 to 2013) and postexpansion (2014 to 2017) cohorts. Demographics, state, and insurance status were abstracted. Outcomes of interest included metastases on presentation and 5-year survival.

**Results:**

Notable decreases were observed in the proportions of patients with bone and soft tissue sarcoma who were uninsured postexpansion in all states. Proportions of patients with Medicaid insurance increased over time in all states but increased more in expansion states. The proportions of all patients with bone and soft tissue sarcoma who had metastases on diagnosis decreased postexpansion, but these changes occurred at similar rates in both expansion and nonexpansion states. Medicaid expansion was also not associated with 5-year overall survival.

**Conclusion:**

Medicaid expansion under the Affordable Care Act did not markedly affect stage at presentation or 5-year OS for bone and soft tissue sarcomas.

Medicaid expansion under the Affordable Care Act took effect on January 1, 2014, and after this, the uninsured rate in the United States decreased from 16.0% in 2010 to 9.1% in 2015.^1^ Under Medicaid expansion, all adults with income levels below 138% of the federal poverty line (FPL) became eligible for Medicaid insurance. The 2012 Supreme Court decision in *National Federation of Independent Business v. Sebelius* determined that states could choose whether to adopt Medicaid expansion. To date, 40 states (80%) have adopted Medicaid expansion and approximately 18.9% of Americans have Medicaid coverage.^2^

Previous work has established that patients with private and Medicaid coverage are more likely than those without insurance to receive preventive care and cancer screening.^3,4^ Uninsured patients are more likely to be diagnosed with late-stage melanoma, prostate cancer, colorectal cancer, and breast cancer.^5,6^ Implementation of the ACA has been associated with improved insurance coverage, higher rates of early-stage diagnosis, and improved mortality for a number of cancers including breast, gynecologic, colorectal, lung, and head/neck.^7,8^ Bone and soft tissue sarcomas represent a rare, heterogenous family of malignancies that necessitates multidisciplinary, often costly care. Limited work has been done thus far investigating clinical outcomes in the setting of Medicaid expansion for patients with bone and soft tissue sarcoma. A 2021 editorial published in *Clinical Orthopaedics and Related Research* urged future work to assess the effect of the ACA on sarcoma patient-centered outcomes, survival, and healthcare costs to evaluate the efficacy of this health policy intervention.^9^

In this study, we sought to answer the following questions: (1) Was the adoption of Medicaid Expansion portion of the ACA on January 1, 2014, associated with earlier stage of diagnosis for patients with bone and soft tissue sarcoma living in states who expanded Medicaid compared with those living in states that did not? (2) Was living in an expansion state in the postexpansion era associated with improved 5-year overall survival (OS) for patients with bone and soft tissue sarcoma?

## Patients and Methods

We queried the National Cancer Database (NCDB) for all patients diagnosed with bone and soft tissue sarcomas from 2010 to 2017. The NCDB is a hospital-based cancer registry that is sourced from 1500 Commission on Cancer-accredited facilities and is organized by the American Cancer Society and the American College of Surgeons. It is the largest cancer registry in the world and captures data on more than 70% of new cancer diagnoses in the United States with 90% 5-year follow-up.^10,11^

We included all adult patients (≥18 years of age at the time of diagnosis) diagnosed with a bone or soft tissue sarcoma between 2010 and 2017. These years were selected so as to begin with implementation of the ACA in 2010 and to allow for 5 years of follow-up data for patients diagnosed in 2017 for survival analyses. Patient age at diagnosis, race, ethnicity, state of residence, and cancer type (bone or soft tissue sarcoma) were captured. Individual insurance status was coded as uninsured, private insurance, Medicaid, Medicare, other government insurance, or unknown. Presence of metastases at diagnosis was used as a surrogate for cancer stage at presentation. Five-year survival data were also abstracted from the database. Of note, we excluded patients with follow-up of less than 1 month from our survival analysis. On review of these patients, almost all had only a single day of follow-up for either a biopsy or pathology review. No additional follow-up or treatment suggests that these patients had suspected sarcoma diagnoses that were then ruled out by biopsy.

Patients were separated into expansion or nonexpansion cohorts based on whether the state in which they resided elected to expand Medicaid on January 1, 2014. States that expanded Medicaid on January 1, 2014, were as follows: Arizona, Arkansas, California, Colorado, Connecticut, Delaware, Hawaii, Illinois, Iowa, Kentucky, Maryland, Massachusetts, Minnesota, Nevada, New Jersey, New Mexico, New York, North Dakota, Ohio, Oregon, Rhode Island, Vermont, Washington, Washington, DC, and West Virginia. Comparisons were made between those diagnosed from 2010 to 2013 (before the expansion of Medicaid on January 1, 2014) compared with those diagnosed from 2014 to 2017 (after the January 1, 2014, Medicaid expansion).

Chi-square tests were used to compare categorical data. Ordinal data comparisons were done using Student t-tests. All tests performed were two-sided, and *P* < 0.05 was considered to be significant.

Pre-post analyses compare outcomes before and after implementation of an intervention, such as a policy change. This analysis strategy is valid only if there are no time-dependent trends that result in improvement of clinical outcomes independent from policy implementation. A difference-in-difference design mitigates this issue by using a comparison group that is subject to the same time-dependent trend but not to the change in policy.^12^ In this study, patients were part of the control group if they resided in a state that did not expand Medicaid on January 1, 2014. Patients were part of the policy intervention group if they resided in a state that expanded Medicaid. Difference-in-difference analyses were done to assess potential changes in the patient insurance status and proportions of patients with metastases on presentation in expansion and nonexpansion states pre-expansion and postexpansion.

Multivariate Cox regression analyses were done separately for bone and soft tissue sarcomas to assess potential factors associated with survival. Variables included in the analysis were demographic parameters, insurance type, living in an expansion state, and diagnosis in the postexpansion era (after January 1, 2014).

All statistical analyses were done using R Statistical Software (version 4.4.2; R Foundation for Statistical Computing, Vienna, Austria).

## Results

### Demographic Data

A total of 43,827 patients in the NCDB were diagnosed with bone or soft tissue sarcomas during the study years of interest (2010 to 2013 and 2014 to 2017; Table 1). In total, 13,638 patients were diagnosed with bone sarcomas and 30,189 patients diagnosed with soft tissue sarcomas. Those diagnosed with bone sarcomas were more likely to be male (χ^2^ = 10.45; *P* = 0.0012) and younger (*P* < 0.0001).

**Table 1:** Demographic Characteristics of Patients With Bone and Soft Tissue Sarcoma in States Who Opted Into Medicaid Expansion on January 1, 2014, Compared With Those Who Did Not

Factor	Bone Sarcoma			Soft Tissue Sarcoma		
	All Patients	Expansion	Nonexpansion	All Patients	Expansion	Nonexpansion
N (%)	13,638	7508 (55.1)	6130 (44.9)	30,189	16,795 (55.6)	13,394 (44.4)
Male, n (%)	7757 (56.9)	4309 (57.4)	3448 (56.2)	16,415 (54.4)	9163 (54.6)	7252 (54.1)
Age, mean (SD)	42.5 (23.5)	43.1 (23.7)	41.8 (23.3)	57.5 (19.6)	57.4 (19.8)	57.7 (19.3)
Race, n (%)						
White	11,217 (82.2)	6197 (82.5)	5020 (81.9)	24,651 (81.7)	13,839 (82.4)	10,812 (80.7)
Black	1341 (9.83)	601 (8.00)	740 (12.1)	3530 (11.7)	1567 (9.33)	1963 (27.1)
Native	70 (0.513)	33 (0.440)	37 (0.604)	123 (0.407)	77 (0.458)	46 (0.343)
Asian/Pacific Islander/Other	4307 (3.16)	393 (5.23)	142 (2.32)	1087 (3.60)	845 (5.03)	242 (1.81)
Unknown	475 (3.48)	284 (3.78)	191 (3.11)	798 (2.64)	467 (2.78)	331 (2.47)
Ethnicity						
Hispanic	1589 (11.7)	948 (12.6)	641 (10.5)	2716 (8.90)	1677 (9.99)	1039 (7.76)
Non-Hispanic	11,707 (85.8)	6397 (85.2)	5310 (86.6)	26,726 (88.5)	14,750 (87.8)	13,394 (89.4)
Other	342 (2.51)	163 (2.17)	179 (2.92)	747 (2.47)	368 (2.19)	379 (2.83)

Among those diagnosed with bone sarcomas, 7508 (55.1%) lived in states that opted into Medicaid expansion on January 1, 2014. In the bone sarcoma cohort, those living in expansion states were older (*P* = 0.0006) with the mean difference of 1.5 years. No differences were observed in proportion of male patients between expansion and nonexpansion states (χ^2^ = 0.885; *P* = 0.347). The proportion of Black patients with bone sarcomas living in expansion states was significantly lower than the proportion of Black patients with bone sarcoma living in nonexpansion states (χ^2^ = 56.2; *P* < 0.0001). A significantly higher proportion of Hispanic patients lived in expansion states compared with nonexpansion states (χ2 = 65.03; *P* < 0.0001).

For those diagnosed with soft tissue sarcoma, 16,795 (55.6%) lived in expansion states. In the soft tissue sarcoma cohort, there were no significant differences in age (*P* = 0.091) or proportion of male patients (χ^2^ = 0.479; *P* = 0.489) between expansion and nonexpansion states. The proportion of Black patients with soft tissue sarcoma living in nonexpansion states was significantly higher than the proportion of Black patients with soft tissue sarcoma living in expansion states (χ^2^ = 179.60; *P* < 0.0001). A significantly higher proportion of Hispanic patients with soft tissue sarcoma lived in expansion states compared with nonexpansion states (χ^2^ = 232.76; *P* < 0.0001).

### Insurance Status

Significant decreases were observed in the proportions of uninsured patients in 2014 to 2017 compared with 2010 to 2013 in both the bone and soft tissue sarcoma groups in both expansion and nonexpansion states, bone expansion (χ^2^ = 9.33; *P* = 0.002); bone nonexpansion (χ^2^ = 13.15; *P* = 0.0003); soft tissue expansion (χ^2^ = 183.64; *P* < 0.0001); soft tissue nonexpansion (χ^2^ = 16.07; *P* < 0.0001); Figure 1). In the difference-in-differences analyses, these changes in proportions between expansion and nonexpansion states were not significantly different for the bone sarcoma cohort (*P* = 0.526) or the soft tissue sarcoma cohort (*P* = 0.162).

**Figure 1:**
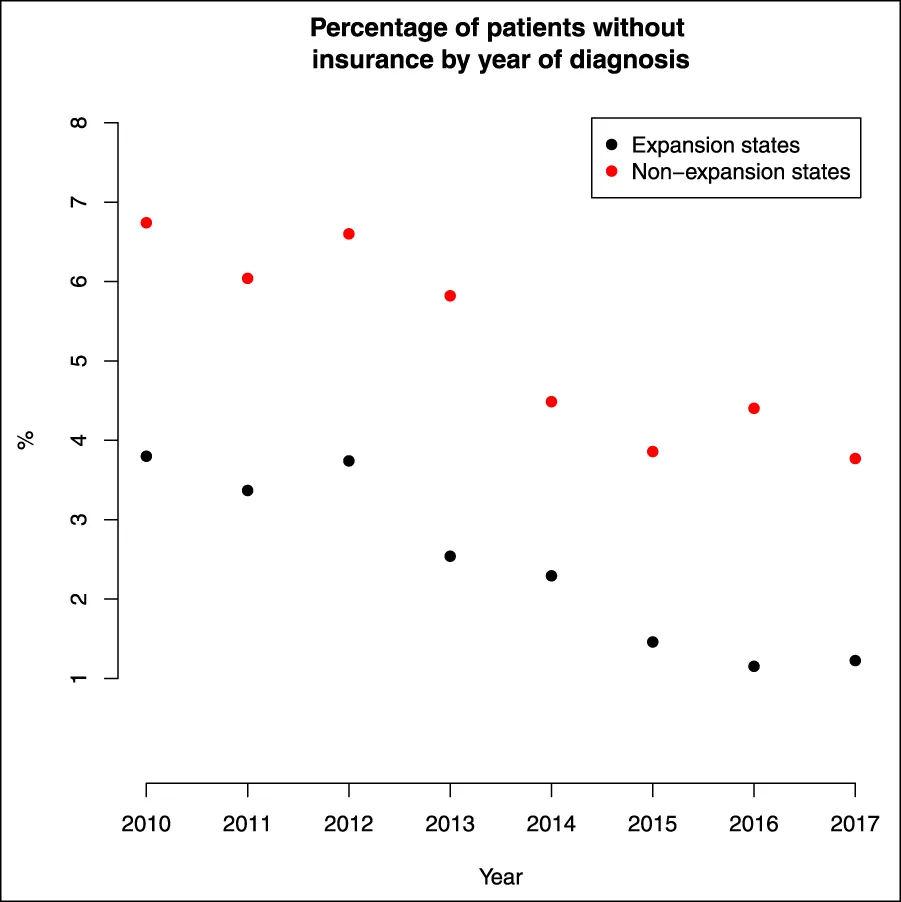
Graph showing that both expansion and nonexpansion states demonstrated similar trends and significant decreases in proportions of uninsured patients over time.

The proportions of patients with Medicaid insurance significantly increased between 2010 to 2013 and 2014 to 2017 in bone and soft tissue sarcoma cohorts in expansion states (bone expansion [χ^2^ = 19.34; *P* < 0.0001]; soft tissue expansion [χ^2^ = 116.54; *P* < 0.0001]). The proportion of patients with Medicaid insurance significantly increased in patients with bone sarcoma in nonexpansion states (χ^2^ = 4.48; *P* = 0.034), but did not change for soft tissue patients in nonexpansion states (χ^2^ = 1.54; *P* = 0.215). The proportion of patients with Medicaid insurance increased more in expansion states compared with nonexpansion states for both bone (*P* < 0.0001) and soft tissue (*P* < 0.0001) cohorts (Table 2).

**Table 2:** Insurance Status at the Time of Presentation for Patients With Bone and Soft Tissue Sarcoma in Expansion States and Nonexpansion States Before and After the January 1, 2014, Medicaid Expansion

Factor	Dx 2010–2013						Dx 2014–2017					
	All		Expansion		Nonexpansion		All		Expansion		Nonexpansion	
	n	%	n	%	n	%	n	%	n	%	n	%
Bone sarcoma												
Uninsured	340	5.23%	118	3.34%	222	7.48%	225	3.15%	61	1.53%	164	5.19%
Private	3511	54.02%	1975	55.93%	1536	51.73%	3855	54.01%	2154	54.16%	1701	53.81%
Medicaid	955	14.69%	548	15.52%	407	13.71%	1124	15.75%	745	18.73%	379	11.99%
Medicare	1333	20.51%	781	22.12%	552	18.59%	1598	22.39%	902	22.68%	696	22.02%
Other government	130	2.00%	45	1.27%	85	2.86%	162	2.27%	58	1.46%	104	3.29%
Unknown	231	3.55%	64	1.81%	167	5.62%	174	2.44%	57	1.43%	117	3.70%
	6500		3531		2969		7138		3977		3161	
Soft tissue sarcoma												
Uninsured	683	4.79%	269	3.41%	414	6.49%	483	3.03%	125	1.40%	358	5.10%
Private	6752	47.33%	3891	49.33%	2861	44.84%	7381	46.36%	4295	48.22%	3086	44.00%
Medicaid	1088	7.63%	689	8.74%	399	6.25%	1467	9.21%	1065	11.96%	402	5.73%
Medicare	5088	35.66%	2803	35.54%	2285	35.82%	5957	37.41%	3203	35.96%	2754	39.26%
Other government	263	1.84%	104	1.32%	159	2.49%	322	2.02%	108	1.21%	214	3.05%
Unknown	393	2.75%	131	1.66%	262	4.11%	312	1.96%	112	1.26%	200	2.85%
	14267		7887		6380		15922		8908		7014	

### Metastases

The proportions of all patients with bone and soft tissue sarcoma who had no metastases on diagnosis increased between 2010 to 2013 and 2014 to 2017, bone (χ^2^ = 18.5; *P* < 0.0001); soft tissue (χ^2^ = 19.1; *P* < 0.0001; Table 3).

**Table 3:** Presence of Metastases on Presentation in Patients With Bone and Soft Tissue Sarcoma in Expansion States and Nonexpansion States Before and After the January 1, 2014, Medicaid Expansion

Factor	Dx 2010–2013						Dx 2014–2017					
	All		Expansion		Nonexpansion		All		Expansion		Nonexpansion	
	n	%	n	%	n	%	n	%	n	%	n	%
Bone sarcoma												
Metastases	999	15.37%	541	15.32%	458	15.43%	1047	14.67%	570	14.33%	477	15.09%
No metastases	4759	73.22%	2572	72.84%	2187	73.66%	5423	75.97%	3020	75.94%	2403	76.02%
Unknown	742	11.42%	418	11.84%	324	10.91%	668	9.36%	387	9.73%	281	8.89%
Soft tissue sarcoma												
Metastases	1199	8.40%	653	8.28%	546	8.56%	1406	8.83%	723	8.16%	638	9.10%
No metastases	11462	80.34%	6260	79.37%	5202	81.54%	12964	81.42%	7206	81.30%	5758	82.09%
Unknown	1606	11.26%	974	12.35%	632	9.91%	1552	9.75%	934	10.54%	618	8.81%

A difference-in-difference linear regression analysis comparing expansion and nonexpansion states demonstrated no notable differences in the changes in proportions of patients presenting with metastases between 2010 to 2013 and 2014 to 2017. This was the case for both bone and soft tissue sarcoma cohorts.

### Mortality

A Cox multivariable regression analysis demonstrated no changes in 5-year OS in expansion states for bone sarcoma (hazard ratio [HR] 0.965; standard error [SE] 0.038; *P* = 0.355). Postexpansion era (2014 to 2017) was not associated with improved survival in our model (HR 0.994; SE 0.042; *P* = 0.89). The combined effect of expansion state and era was also not significant (HR 0.923; SE 0.057; *P* = 0.156), demonstrating that there was no significant effect of time interval (pre-expansion vs postexpansion) in either the expansion or nonexpansion states. A Kaplan-Meier curve for bone sarcoma 5-year survival is shown in Figure 2.

**Figure 2:**
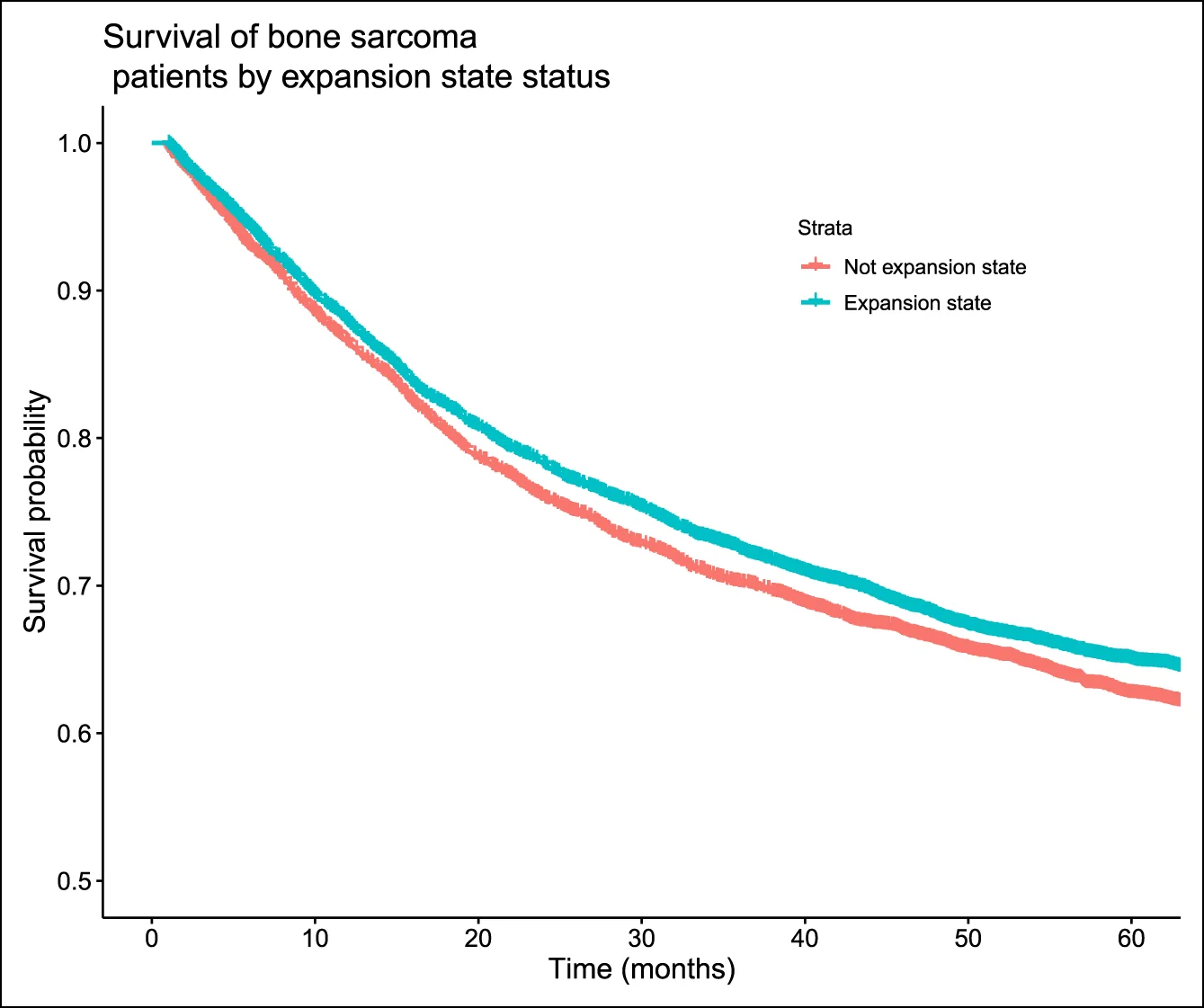
Graph showing a Kaplan-Meier curve demonstrating 5-year overall survival for patients with bone sarcoma in expansion (blue) and nonexpansion (red) states.

A Cox multivariable regression analysis of patients with soft tissue sarcoma demonstrated improved 5-year OS in expansion states in general (HR 0.931; SE 0.026; *P* = 0.0077). However, similar to bone sarcoma, postexpansion era (2014 to 2017) was not associated with improved survival (HR 1.006; SE 0.029; *P* = 0.829), nor was the combined effect of expansion state and era (HR 0.986; SE 0.039; *P* = 0.7165). A Kaplan-Meier curve for soft tissue sarcoma 5-year survival is shown in Figure 3.

**Figure 3:**
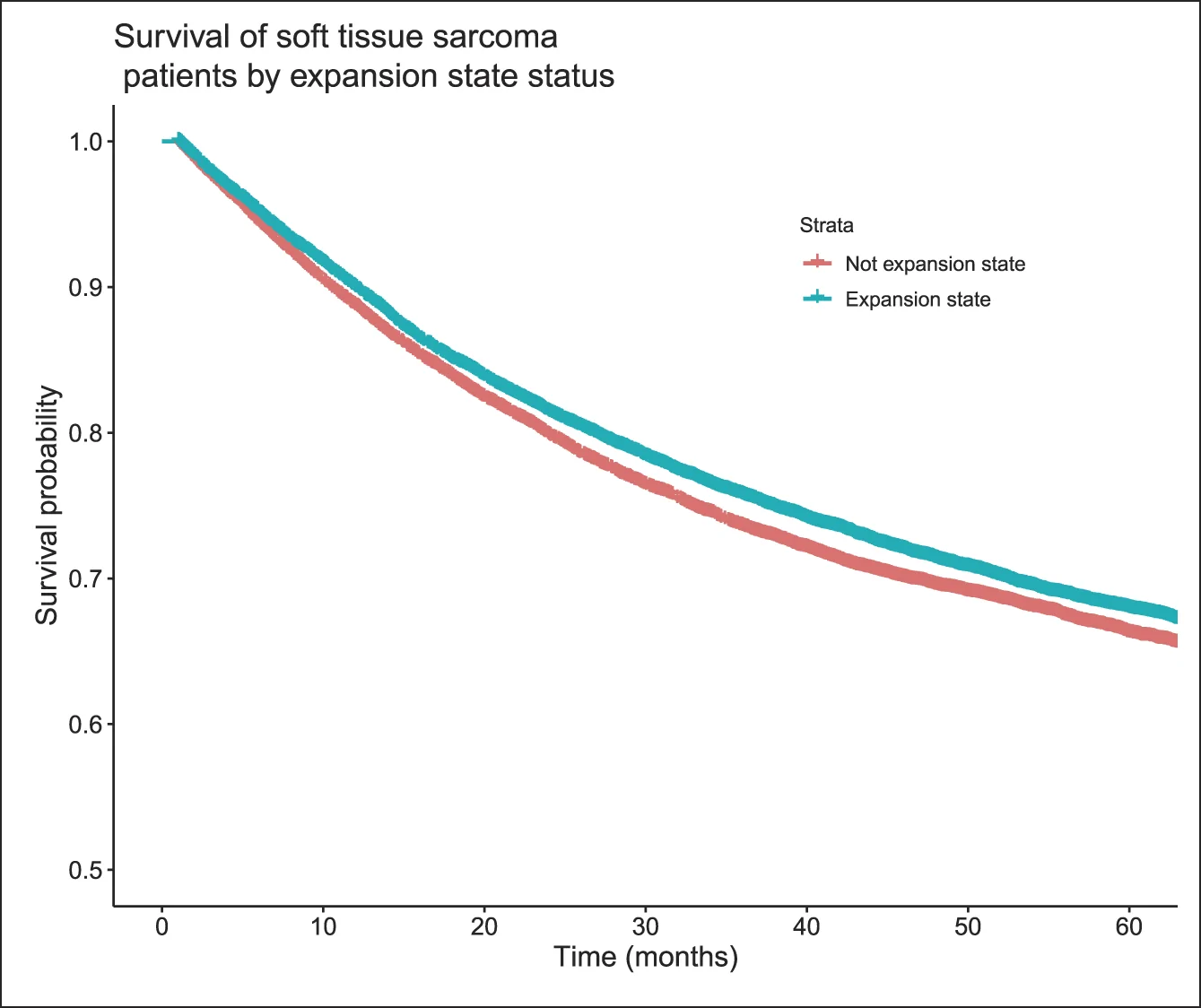
Graph showing a Kaplan-Meier curve demonstrating 5-year overall survival for patients with soft tissue sarcoma in expansion (blue) and nonexpansion (red) states.

## Discussion

In our analysis of 43,827 patients with bone and soft tissue sarcomas within the National Cancer Database (NCDB), we found that the adoption of the Medicaid expansion on January 1, 2014, as part of the Affordable Care Act (ACA) was not associated with changes in proportions of uninsured patients with bone and soft tissue sarcoma. Although we found notable decreases in proportions of patients with uninsured sarcoma over time, these decreases occurred in both expansion and nonexpansion states. Proportions of patients with Medicaid insurance increased over time in expansion and nonexpansion states but increased more in expansion states. We found that Medicaid expansion was not associated with changes in cancer stage at presentation or 5-year overall survival (OS). Proportions of all patients with bone and soft tissue sarcoma who had metastases on diagnosis decreased over time in expansion and nonexpansion states. No changes were observed over time in 5-year overall survival (OS) for bone sarcoma in expansion and nonexpansion states. Although patients with soft tissue sarcoma demonstrated improved 5-year OS in expansion states in general, postexpansion era was not associated with improved survival.

Our finding of decreased proportions of uninsured patients with cancer after the implementation of the ACA has been corroborated in the literature. Jemal *et al* investigated insurance status and stage at diagnosis of 1.8 million patients with newly diagnosed cancer within the NCDB between 2011 and 2014. Between pre-ACA and post-ACA periods, the percentage of uninsured patients with newly diagnosed cancer decreased in both Medicaid expansion and nonexpansion states.^13^ Similarly, Han and coauthors studied 2.4 million patients with cancer in the North American Association of Central Cancer Registries (NAACCR) and found decreases in the percentages of uninsured patients with cancer in all states after the implementation of the ACA with the greatest decreases occurring in Medicaid-expansion states.^14^ Malik and coauthors studied ∼15,0000 patients with bone and soft tissue sarcomas in the Surveillance, Epidemiology, and End Results (SEER) database from 2007 to 2015. Within states that adopted the Medicaid expansion in 2014, the authors found an increase in the proportion of sarcoma patients with Medicaid insurance and a decrease in the proportion of uninsured patients.^15^ We similarly found postexpansion increases in proportions of bone and soft tissue sarcoma patients with Medicaid insurance, particularly in expansion states.

Despite improvements in insurance coverage for patients with sarcoma after the ACA expansion, we found that Medicaid expansion was not associated in improvements in stage at presentation or in 5-year OS. Although our findings demonstrate improvements in stage at presentation for patients with bone and soft tissue sarcoma and in OS for patients with soft tissue sarcoma over time, but our analysis suggests that this is independent from Medicaid expansion. Malik similarly found improvements over time in early-stage diagnosis for sarcoma, but different from our study, found notable increases in early-stage diagnosis for primary bone sarcoma only in expansion compared with nonexpansion states.^15^

Insurance status has been shown to have important implications for outcomes for patients with sarcoma. Smartt et al studied 12,000 sarcoma patients within the SEER database and found that Medicaid insurance was associated with distant metastases at diagnosis and reduced disease-specific survival even after accounting for stage at presentation. The authors posit that these findings may be due to a variety of complex sociodemographic factors.^16^ Medicaid insurance has been associated with higher rates of metastases on presentation, lower rates of neoadjuvant or adjuvant radiation therapy, and reduced overall survival for sarcoma in several other studies.^17-22^ Minimizing time from diagnosis to treatment is important for patients with sarcoma,^23^ and Medicaid patients with sarcoma have longer time to treatment initiation compared with patients with commercial insurance.^24,25^ Possibly, we found few improvements in stage at presentation and 5-year OS for patients with sarcoma despite improved insurance coverage after Medicaid expansion due to factors such as these.

Poor outcomes in patients with Medicaid insurance have been corroborated for other cancer types.^26-28^ Medicaid expansion has been associated with small improvements in early-stage diagnosis and mortality for colorectal, lung, melanoma, female breast, and pancreatic cancer for those residing in expansion states compared with those in nonexpansion states in some studies.^7,8,13^ However, other studies have found that although early-stage diagnosis for many cancers seems to be improving with time, there are no differences in early diagnosis for most cancers between expansion and nonexpansion states.^14^ In this study, we similarly demonstrate improvements in sarcoma stage at presentation over time with no differences between expansion and nonexpansion states.

The strength of this study is that it investigates a large national database with good patient capture and excellent follow-up. The utilization of a large and longitudinal national database allows for the study of the implications of policy interventions such as the ACA which is often challenging in smaller, institutional cohorts. However, it has weaknesses inherent to cancer database studies. Although the NCDB is regularly audited for completeness and accuracy, it is subject to errors and incompleteness in coding which could potentially influence our results.

We also collected insurance status only at the time of diagnosis. Possibly, this insurance status changed over the course of treatment, which may affect our results. In addition, we investigated state of residence at time of diagnosis. This does not account for patients moving during their treatment period, nor does it account for the fact that patients with sarcoma often travel and occasionally relocate for treatment.

Although Medicaid expansion began on January 1, 2014, there was an expected lag in both states enrolling in the Medicaid expansion and in patients themselves enrolling in the associated programs. We used a difference-in-difference study design to account for this, but this design does not fully account for the realities of delays in enrollment.

Finally, only the recorded factors within the database are available for analysis, limiting the variables we could consider. Although the NCDB captures approximately 70% of new cancer diagnoses in the United States, it is prone to selection bias. The NCDB is not population-based, and it only captures data from Commission on Cancer-accredited facilities, which may limit sampling of patients. It is therefore possible that we are missing sarcoma diagnoses that might affect our results, particularly from underresourced hospitals. In addition, the NCDB only includes certain variables, and there is a lack of granularity in variables pertaining to exact diagnoses, cancer progression/recurrence, and other more nuanced details of treatment and outcomes. Although overall survival is reported, there is no way to determine disease-specific survival within the database. This limited the outcomes that we could evaluate within this study.

## Conclusion

The post-2014 era after Medicaid expansion of the ACA is associated with decreased rates of uninsured patients in both expansion and nonexpansion states and with a notable increase in proportions of Medicaid patients in expansion states. Improvements were observed in cancer stage at diagnosis for both patients with bone and soft tissue sarcoma observed over time, but these improvements were seen in all states studied and were not specifically associated with expansion state status. The Medicaid expansion is not itself associated with improved 5-year overall survival in either bone or soft tissue sarcoma. Additional research is needed to better understand how to best identify early and support patients with sarcoma, particularly those who are uninsured or have Medicaid insurance.
